# A New Human Perception-Based Over-Exposure Detection Method for Color Images

**DOI:** 10.3390/s140917159

**Published:** 2014-09-15

**Authors:** Yeo-Jin Yoon, Keun-Yung Byun, Dae-Hong Lee, Seung-Won Jung, Sung-Jea Ko

**Affiliations:** 1 School of Electrical Engineering, Korea University, 145, Anam-ro, Seongbuk-gu, Seoul 136-701, Korea; E-Mails: yjyoon@dali.korea.ac.kr (Y.-J.Y.); kybyun@dali.korea.ac.kr (K.-Y.B.); dhlee@dali.korea.ac.kr (D.-H.L.); 2 Department of Multimedia Engineering, Dongguk University-Seoul, 30 Pildong-ro 1-gil, Jung-gu, Seoul 100-715, Korea; E-Mail: swjung83@dongguk.edu

**Keywords:** over-exposure, over-exposure correction, over-exposed region detection, saturation sensitivity, human visual system

## Abstract

To correct an over-exposure within an image, the over-exposed region (OER) must first be detected. Detecting the OER accurately has a significant effect on the performance of the over-exposure correction. However, the results of conventional OER detection methods, which generally use the brightness and color information of each pixel, often deviate from the actual OER perceived by the human eye. To overcome this problem, in this paper, we propose a novel method for detecting the perceived OER more accurately. Based on the observation that recognizing the OER in an image is dependent on the saturation sensitivity of the human visual system (HVS), we detect the OER by thresholding the saturation value of each pixel. Here, a function of the proposed method, which is designed based on the results of a subjective evaluation on the saturation sensitivity of the HVS, adaptively determines the saturation threshold value using the color and the perceived brightness of each pixel. Experimental results demonstrate that the proposed method accurately detects the perceived OER, and furthermore, the over-exposure correction can be improved by adopting the proposed OER detection method.

## 1. Introduction

A dynamic range indicates the ratio between the largest and smallest light intensities measurable. While the dynamic range of a real scene ranges from 10^5^:1 to 10^9^:1, that of the digital camera is about 10^3^:1. Thus, both under- and over-exposure problems may occur in photographs when the camera captures a scene having a range of light exceeding the dynamic range of the camera sensor [1,2]. In particular, the over-exposure problem occurs when the camera captures a scene in which the light intensity is too strong to be captured by the camera. As a result, an over-exposed region (OER) characterized by a high brightness, low chromaticity, and loss of detail can appear in the captured photograph. To solve the over-exposure problem, various techniques which expand the dynamic range of image sensors [3–5] or modify the operating system of image sensors [6] have been employed in image capture.

In the field of image processing, many restoration methods have been proposed to improve the visibility of the over-exposed image by correcting the OER within the image [7–14]. These methods first detect, and then correct, the OER in the image. Specifically, OER detection is performed by finding a region consisting of pixels with a high level of brightness and low chromaticity. For example, because pixels with high brightness levels tend to have low chromaticity as well, several previous methods adopt the use of simple brightness thresholding [7–9]. If the brightness value of a pixel is larger than a predefined threshold value, the pixel is identified as over-exposed. On the other hand, Guo's method [10] obtains an over-exposed map using both the brightness and color values of the pixels in the CIELAB color space. The OER is then detected by thresholding the over-exposed map. In addition, some methods [11–13] estimate the OER by thresholding each RGB color channel separately. Thus, in these methods, the detected OER is much larger than that obtained by the previously described detection methods.

Although the aforementioned methods can roughly identify the OER, the detection result often deviates from the actual OER perceived by the human eye. In other words, the OER detected by conventional methods can be smaller than the perceived OER or include regions which do not seem to be over-exposed. Furthermore, since inaccurate detection may degrade the restoration performance, detecting an accurate OER is an important issue for over-exposure correction.

In this paper, we introduce a novel OER detection method that accurately identifies the perceived OER in the image. Based on the observation that the sensitivity of the human visual system (HVS) to the over-exposure is dependent on the color and saturation, as well as the brightness, we newly develop an adaptive saturation thresholding method for detecting the perceived OER. In particular, we design a new function for determining a saturation threshold value using the hue and value channels of the hue-saturation-value (HSV) color space. The experimental results demonstrate that the proposed method more accurately detects the perceived OER than conventional methods, and that accurate OER detection can lead to the improved OER correction performance. To the best of our knowledge, the proposed method is the first method to use the saturation sensitivity characteristics of the HVS for detecting the OER.

The remainder of this paper is organized as follows: conventional OER detection methods and their limitations are presented in Section 2. In Section 3, the saturation sensitivity of the HVS is described. The proposed method is introduced in Section 4, and the experimental results are given in Section 5. Section 6 provides some concluding remarks.

## 2. Limitations of Conventional OER Detection Methods

In this section, we briefly describe representative conventional OER detection methods and their limitations.

(1) Brightness thresholding method
  In [8,9] pixels with high brightness values are identified as over-exposed. In [8], brightness thresholding is performed in the CIELAB color space. If the brightness value L* of a pixel is larger than 98, where 0 ≤ L* ≤ 100, the pixel is over-exposed. In [9], if the Y value in a YUV color space is greater than or equal to 230, the pixel is detected as over-exposed.
(2) Over-exposed map based thresholding method
  In [10], the use of an over-exposed map is proposed to describe how much a particular pixel is affected by an over-exposure. Because the OER generally has a high level of brightness and low chromaticity, the over-exposed map for pixel *i*, *i.e.*, *M_i_*, is defined as follows:
  (1) $$M_{i}=\frac{1}{2}\cdot (\tanh(\frac{1}{60}\cdot ((L_{i}-80)+(40-{\left\|C_{i}\right\|}_{2})))+1)$$
  where *L_i_* and *C_i_* indicate L* and [a* b*]^T^ of pixel *i* in the CIELAB color space, respectively. The pixel is more likely to be over-exposed if *L_i_* is larger or if ‖*C_i_*‖_2_ is smaller. In [8,10,14], using an over-exposed map, pixels with *M_i_* ≥ 0.5 are identified as over-exposed.

**Figure 1.**
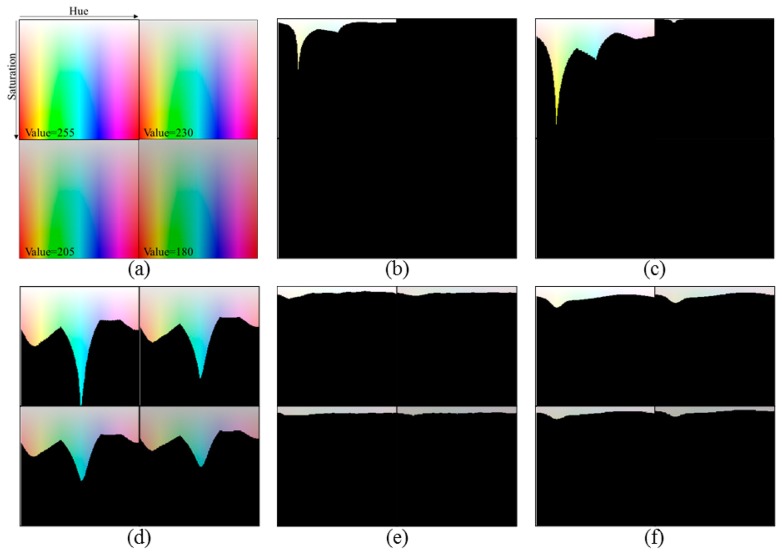
Comparison of the OER detection results. (**a**) Original test image; (**b**) OER detected using the brightness thresholding method (L* > 98) [8]; (**c**) OER detected using the brightness thresholding method (Y ≥ 230) [9]; (**d**) OER detected using the over-exposed map based thresholding method [10]; (**e**) Perceived OER identified through a subjective evaluation; (**f**) OER detected using the proposed method.

To evaluate the performance of the OER detection methods, we used the test image shown in Figure 1a. In the test image, the hue (*H*) in the HSV color space varies along the horizontal line, and the saturation (*S*) varies along the vertical line, whereas the value (*V*) of all pixels is equal to 255, 230, 205, and 180 in the upper-left, upper-right, lower-left, and lower-right sub-images, respectively.

Figure 1b–d shows the detection results obtained by conventional OER detection methods. The colored region indicates the detected OER, and the black region is a well-exposed region (WER) identified by the method used. When we detect the region in which the L* values of the pixels are larger than 98 [8], a small region around the pure white and bright yellow colors is detected as an OER only in the case of *V* = 255, as shown in Figure 1b. In Figure 1c, pixels whose Y ≥ 230 are detected [9]. When *V* = 255, the detected OER includes yellow and cyan colors, which do not seem to be over-exposed. A very small OER for yellow is detected when *V* = 230, and the OER is not generated when *V* = 205 or 180. On the other hand, the over-exposed map based thresholding method intentionally detects a wide OER for OER correction, as shown in Figure 1d [10]. Thus, overly large regions are identified as OERs even though the value of *V* decreases. In addition, more regions for cyan and blue are considered to be over-exposed compared to the other colors, which does not coincide with the perceived OER.

We can conclude from the above observation that the conventional methods detect the OER roughly; however, the results largely deviate from the perceived OER. Although the YUV and CIELAB color spaces are designed by considering the HVS, simply thresholding the brightness value or the over-exposed map is insufficient for accurately detecting the perceived OER. In addition, we found that the inaccurate detection result can degrade the OER restoration performance, which will be shown in Section 5. Thus, a more enhanced detection method is required for effective OER correction. To this end, in this paper, we propose a novel OER detection method that can identify the perceived OER accurately considering the HVS. Based on the characteristics of a colorimetric purity discrimination as well as the brightness perception of the human eye, we design a new adaptive saturation thresholding method. Prior to introduction of the proposed OER detection method, we describe the colorimetric purity discrimination of the human eye in the next section.

## 3. Saturation Sensitivity of HVS

In [15–19], the saturation sensitivity of the human eye is characterized through the colorimetric purity discrimination, which describes the minimum amount of spectral light, *P_c_*, that allows a mixture of spectral light and white to be distinguished from white alone [15]. Mathematically, *P_c_* can be defined as follows:

(2) $$P_{c}=\frac{L_{\lambda }}{L_{w}+L_{\lambda }}$$

where *L_λ_* is the luminance of the spectral color, and *L_w_* is the luminance of white. Here, *P_c_* is also called the least colorimetric purity.

**Figure 2.**
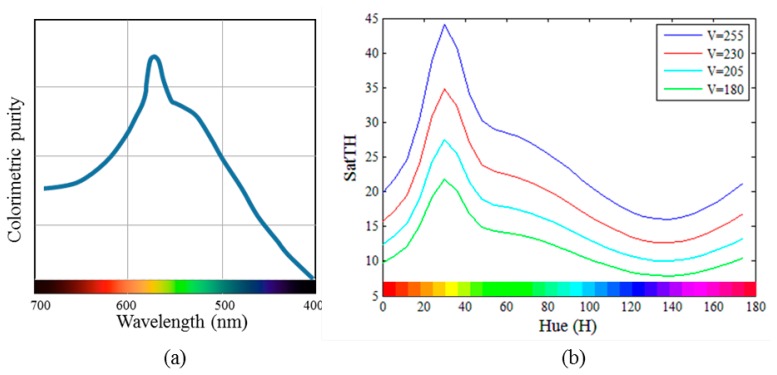
(**a**) The least perceptible colorimetric purity [16]; (**b**) Saturation threshold values, *S_TH_*, calculated using the proposed Function (3).

Figure 2a shows the least perceptible colorimetric purity plotted as a function of wavelength [16]. The data reported in [17–19] also show similar trends. As shown in Figure 2a, the values of *P_c_* vary depending on the wavelength. Specifically, *P_c_* has the maximum value near 570 nm and decreases at the ends of the spectrum. Therefore, a high luminance of the spectral light is required for yellow to be distinguished from pure white; however, for other colors such as red, blue, and violet, a mixture of white and a slight amount of spectral light can be easily distinguished from pure white. In other words, in terms of visual perception, yellow has low saturation power, whereas red, blue, and violet have relatively high saturation power. In addition, it was also reported in [18] that the wavelength sensitivity of the purity discrimination decreases as the luminance decreases.

## 4. Proposed Method

### 4.1. Simulation

**Figure 3.**
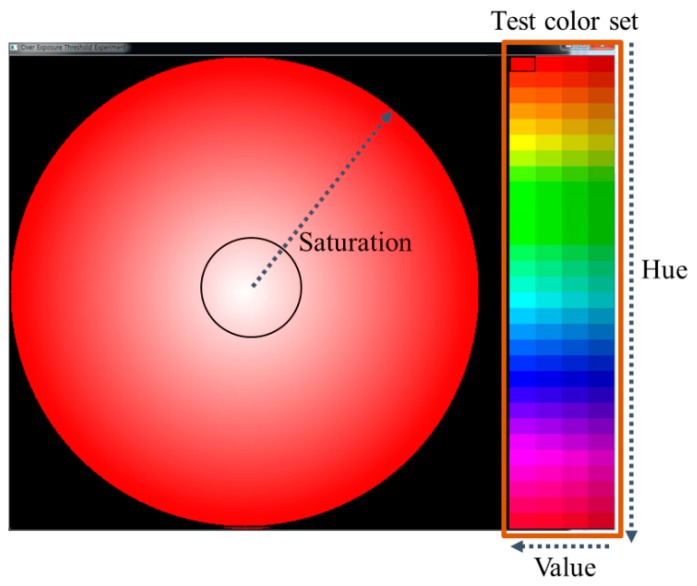
Test set for evaluating the saturation sensitivity of HVS.

To confirm that the aforementioned saturation sensitivity of the HVS is related to the perceived OER detection, we performed an experiment using the test set shown in Figure 3. We design the test set using the HSV color space. Within the large circle in Figure 3, the value of *S* varies from 0 to 255 along the radius of the circle for each color in the test color set. The test color set is composed of 120 colors having four *V* values between 180 and 225, and sixty *H* values between 0° and 180°. The test set was displayed using a 27-in Samsung TA950 monitor under a measured environment illuminance of about 750 lux. The monitor has a peak brightness of 300 cd/m^2^ and a color temperature of 6500 K. The viewing distance was set to 80 cm from the screen. Twelve non-experts participating as subjects of the experiment were asked to make a small circle for the region appeared as pure white by adjusting the length of its radius, as shown in Figure 3. We then collected the *S* values for the positions on which the small circle is placed. Note that the obtained *S* value can be a subjective criterion for classifying the OER and WER within the large circle.

Using the results of this experiment, we detected the OER for the image in Figure 1a. As Figure 1e shows, by following the trend in the wavelength saturation power, the vertical position classifying the OER and WER varies along with the value of *H*. Specifically, the values of criterion *S* for yellow are larger than those for the other colors. In addition, the values of criterion *S* are gradually reduced while the value of *V* decreases. It can therefore be noted that the perceived OER detection is closely related to the aforementioned saturation sensitivity of the HVS. Accordingly, we propose a method for detecting the perceived OER using the saturation information of each pixel. In other words, we detect the perceived OER by adaptively thresholding the saturation value with consideration for the color and brightness of each pixel. Based on the saturation sensitivity of the HVS and our subjective evaluation result, a method for calculating the saturation threshold value is proposed in the following section.

### 4.2. Adaptive Saturation Threshold Value Selection Scheme

Using the characteristics of the saturation sensitivity of the HVS, we first propose the use of an adaptive saturation threshold value selection scheme. As mentioned above, because the saturation power of yellow is relatively low, the saturation threshold value should be large enough to detect the perceived OER occurring in the yellow region. On the other hand, the saturation threshold values for red and violet should be smaller than the others since the saturation power for these colors is much higher. In this manner, we can accurately detect the perceived OER using the color-dependent saturation threshold values based on the trend in the saturation sensitivity.

A new method for calculating the saturation threshold value using the components in the HSV color space is presented. Using a MATLAB curve fitting toolbox, we fitted the data obtained from our subjective evaluation with the product of two Gaussian mixture models (GMMs). The product of the GMMs was adopted mainly from our observation that the hue and value channels were weakly correlated and the empirical distribution had few smoothed peaks. The proposed function for calculating the saturation threshold value, *S_TH_*, is given as follows:

(3) $$\begin{array}{l} S_{TH}=F_{v}(v)\cdot F_{h}(h)\\ F_{v}(v)=\exp(\frac{2.4\cdot (v-255)}{255})\\ F_{h}(h)=17\cdot \exp(-{\left(\frac{h-30}{11.46}\right)}^{2})+30015\cdot \exp(-{\left(\frac{h-136.3}{100.1}\right)}^{2})-29999\cdot \exp(-{\left(\frac{h-136.3}{100}\right)}^{2}) \end{array}$$

where *v* and *h* indicate the *V* and *H* values in the HSV color space, respectively. Here, for an 8-bit image, *h* is represented by values of 0° to 180°, and a value of *v* between 180 and 255 is only considered based on our empirical observation that the OER does not exist when *v* is smaller than 180. The first term, *F_v_*(*v*), is a scaling factor for adjusting the value of *S_TH_* according to *v*. As *v* decreases, *S_TH_* is also decreased, and *vice versa*. The second term controls the value of *S_TH_* according to *h* by following the trend in saturation sensitivity of the HVS. In *F_h_*(*h*), the first Gaussian function generates a peak around the yellow colors, and the varying trend for the other colors is produced by the sum of the second and third Gaussian functions. An R-squared value (R^2^), which indicates how closely the estimated model fits to the actual data in range from 0 to 1 [20], of Equation (3) is obtained as approximately 0.86. The graph of Equation (3) is shown in Figure 2b, which indicates the *S_TH_* values for four different values of *v* and 60 values of *h*. Note that the proposed model for the saturation threshold value has a very similar form as the saturation sensitivity of the HVS shown in Figure 2a.

### 4.3. Proposed Over-Exposed Region Detection Method

As described above, in order to calculate the saturation threshold, the *H* and *V* values of each pixel are used in Equation (3). However, in natural images, the surrounding context affects the over-exposure perception of the HVS, and thus the saturation threshold derived from the simple experimental setup as shown in Figure 3 is not directly applicable. To this end, correlations of hue, brightness, and saturation and even their mutual correlations need to be considered within the surrounding context. Among many possible correlations, the brightness perception with respect to the surrounding context is most extensively studied since the brightness is the dominant element of visual contrast and largely determines the visual perception. We thus consider the perceived brightness instead of the naive brightness when obtaining the saturation threshold *S_TH_* from real-world images. Specifically, we obtain the perceived brightness image *Ṽ* by applying a Retinex-based adaptive filter [21] to the brightness image *V*. In [21], the Retinex-based adaptive filter is applied to the luminance channel only for rendering the local adaptation of the human eye based on the Retinex theory [22]. Likewise, by applying this scheme to the *V* image, we estimate the locally adapted perceived brightness [23]. More details about mathematical computation can be found in Section III-B: Local Adaptation in [21]; however, any other method of estimating the perceived brightness can be substituted.

**Figure 4.**
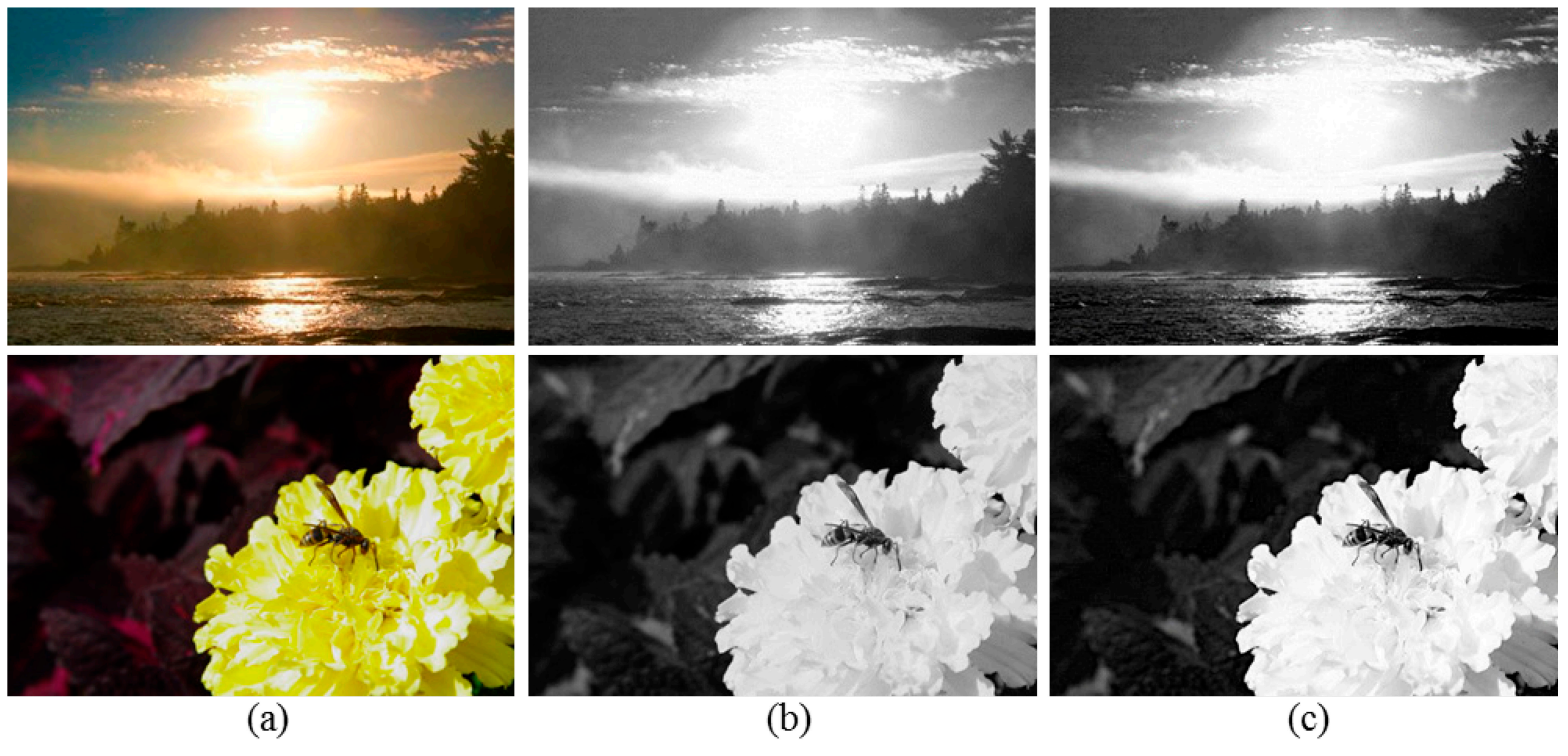
Perceived brightness estimation results. (**a**) Original images; (**b**) The *V* images of (a); (**c**) The perceived *V* images of (a).

Figure 4 shows the *V* images of the original images and their corresponding *Ṽ* images, respectively. As shown in Figure 4c, the brightness perceived by the human eye can be estimated by considering the correlation with surrounding pixels in image. Accordingly, in the proposed method, the perceived OER is detected based on both the saturation and brightness perception characteristics of the HVS.

Finally, the proposed OER detection is performed as follows. For pixel *p*, the following steps are conducted:

Step 1 Convert the R, G, and B values into *H*, *S*, and *V* values.
Step 2 Calculate the perceived brightness value *Ṽ* by applying the Retinex-based adaptive filter [21] to the *V* value.
Step 3 Calculate *S_TH_* using Equation (3) with the *H* and *Ṽ* values.
Step 4 Compare the *S_TH_* and *S* values. If *S* < *S_TH_*, *p* is an over-exposed pixel. If *S* ≥ *S_TH_*, *p* is a properly exposed pixel.

In the case of R = G = B, because the hue is undefined and the saturation is always equal to zero, the saturation thresholding method cannot be applied. Thus, in this case, if the value of *Ṽ* is equal to or larger than 230, the pixel is detected as over-exposed.

For the test image in Figure 1a, the perceived OER detected by the proposed method is shown in Figure 1f. As the results show, the proposed method detects the OER similarly to that recognized by the human eye, as shown in Figure 1e.

## 5. Experimental Results

### 5.1. Comparison of the Over-Exposed Region Detection Results

**Figure 5.**
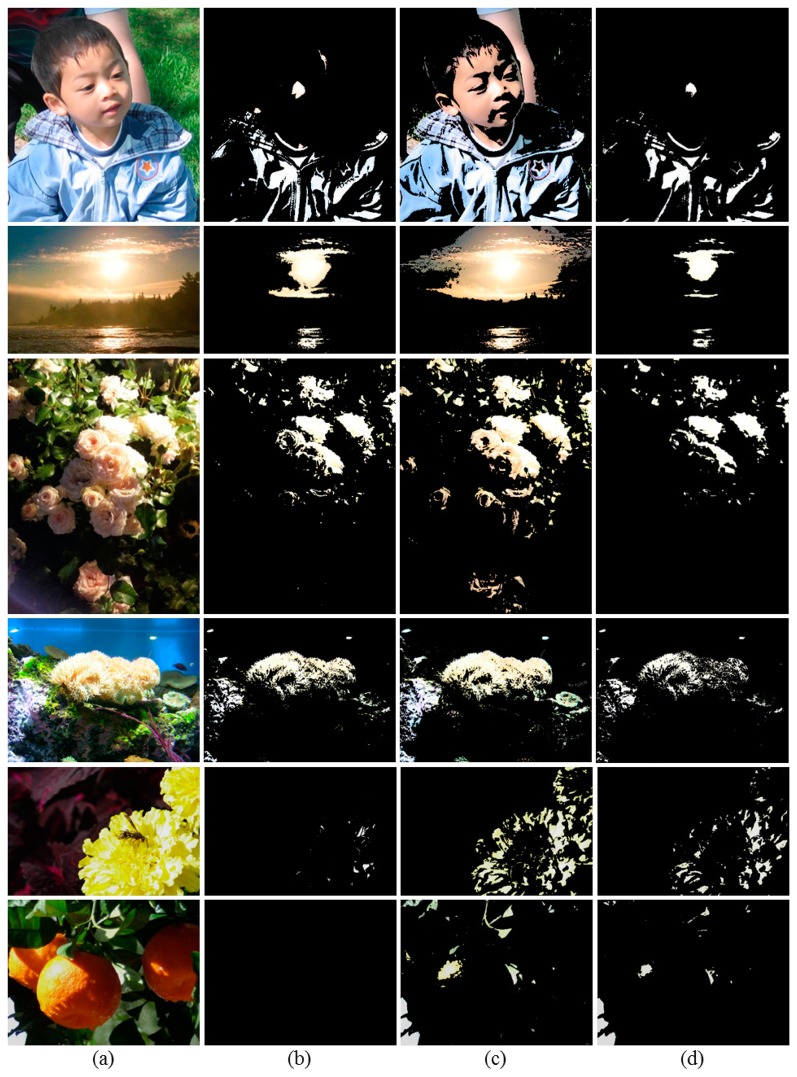
Comparison of OER detection results. (**a**) Original *Kid*, *Sky*, *Roses*, *Coral*, *Flower and bee*, and *Oranges* images; (**b**) OERs detected using the brightness thresholding method (Y ≥ 230) [9]; (**c**) OERs detected using the over-exposed map based thresholding method [10]; (**d**) Perceived OERs detected using the proposed method.

To demonstrate the effectiveness of the proposed method, we first compare the OER detection results obtained by the conventional brightness thresholding method [9], over-exposed map based thresholding method [10], and the proposed method in Figure 5.

Because the over-exposed map based thresholding method over-detects the OER and shows a particularly low level of accuracy for cyan as shown in Figure 1d, the detected OERs shown in Figure 5c are much larger than the other detection results and contain WERs having noticeable colors. In particular, for the *Kid* image, note that the nose and center and right regions of the blue jumper are mainly over-exposed. However, most regions of the face, arm, and jumper are identified as the OER by the over-exposed map based thresholding method. In the detection result of the *Sky* image in Figure 5c, besides the highlighted area in the sky, regions having noticeable blue and sunset yellow colors are also identified as the OER, which significantly deviates from the perceived OER.

For the test images except the *Flower and bee* and the *Oranges* images, the brightness thresholding method provides better detection results compared with the over-exposed map based thresholding method as shown in Figure 5b; however, it also tends to detect a wider region than the perceived OER. On the other hand, in the *Flower and bee* image, it can be seen that petals of the yellow flower have a white or severely faded yellow color in certain areas, and we can therefore recognize that they are over-exposed. However, as shown in Figure 5b, the brightness thresholding method can barely detect the perceived OER because the Y values of most of these regions are actually close to 220. Likewise, the perceived OER in the *Oranges* image is never detected by the brightness thresholding method.

On the other hand, we can observe from Figure 5d that the perceived OERs are clearly detected by the proposed method for all images. As an example, for the *Coral* image, the conventional methods identify most of the coral as an OER. However, even though the coral has an ivory color with a high level of brightness, not all of the coral region seems to be over-exposed, and a partial region in the right area of the coral seems to be properly exposed. Using the proposed method, the OER as perceived by the HVS is accurately detected.

**Figure 6.**
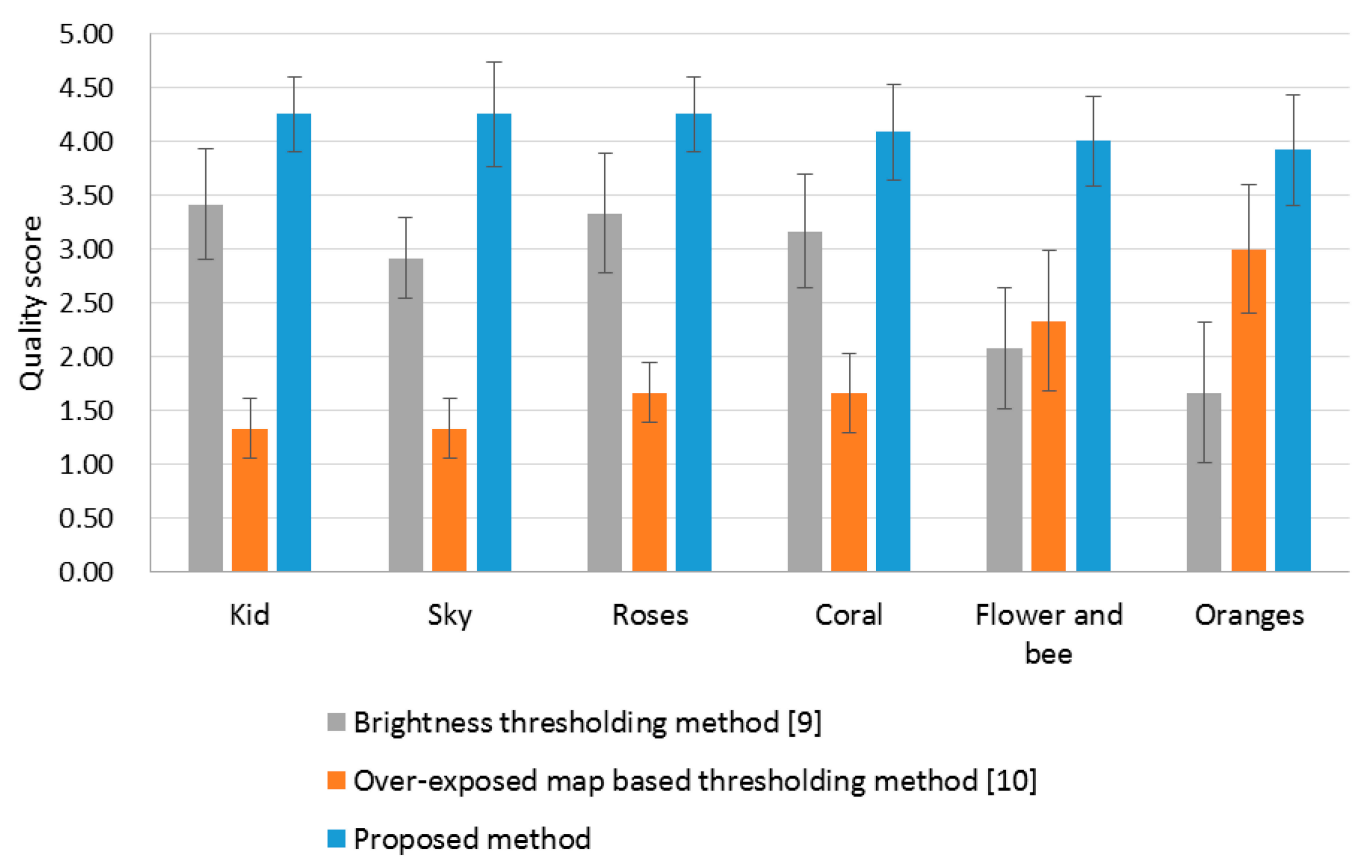
Subjective evaluation result for the OER detection results.

We perform the subjective quality evaluation of the OER detection results. Under the same experimental environment described in Section 4.1, three detection result images were displayed simultaneously with the original over-exposed image. Twelve subjects (mean age = 27.4 years; range = 24 to 29 years; one female and eleven males) were asked to assess the detection accuracy by marking the comparison scale according to Table 1 [24]. As shown in Figure 6, the average subjective scores with 95% confidence interval reveal that the proposed method detects the OER very close to what the human eye perceives and outperforms the conventional methods.

### 5.2. Comparison of Correction Results

The performance of existing OER correction methods may occasionally be degraded owing to improper OER detection. Thus, in this section, we show that the performance of OER correction can be improved by adopting the proposed OER detection method. For comparison, OERs were detected using the brightness thresholding method [9], the over-exposed map based thresholding method [10], and the proposed method. The detected OERs were corrected using Guo's method [10]. We simulated the correction method using Matlab, and the parameter values and details unspecified in [10] were determined experimentally for the best performance.

**Figure 7.**
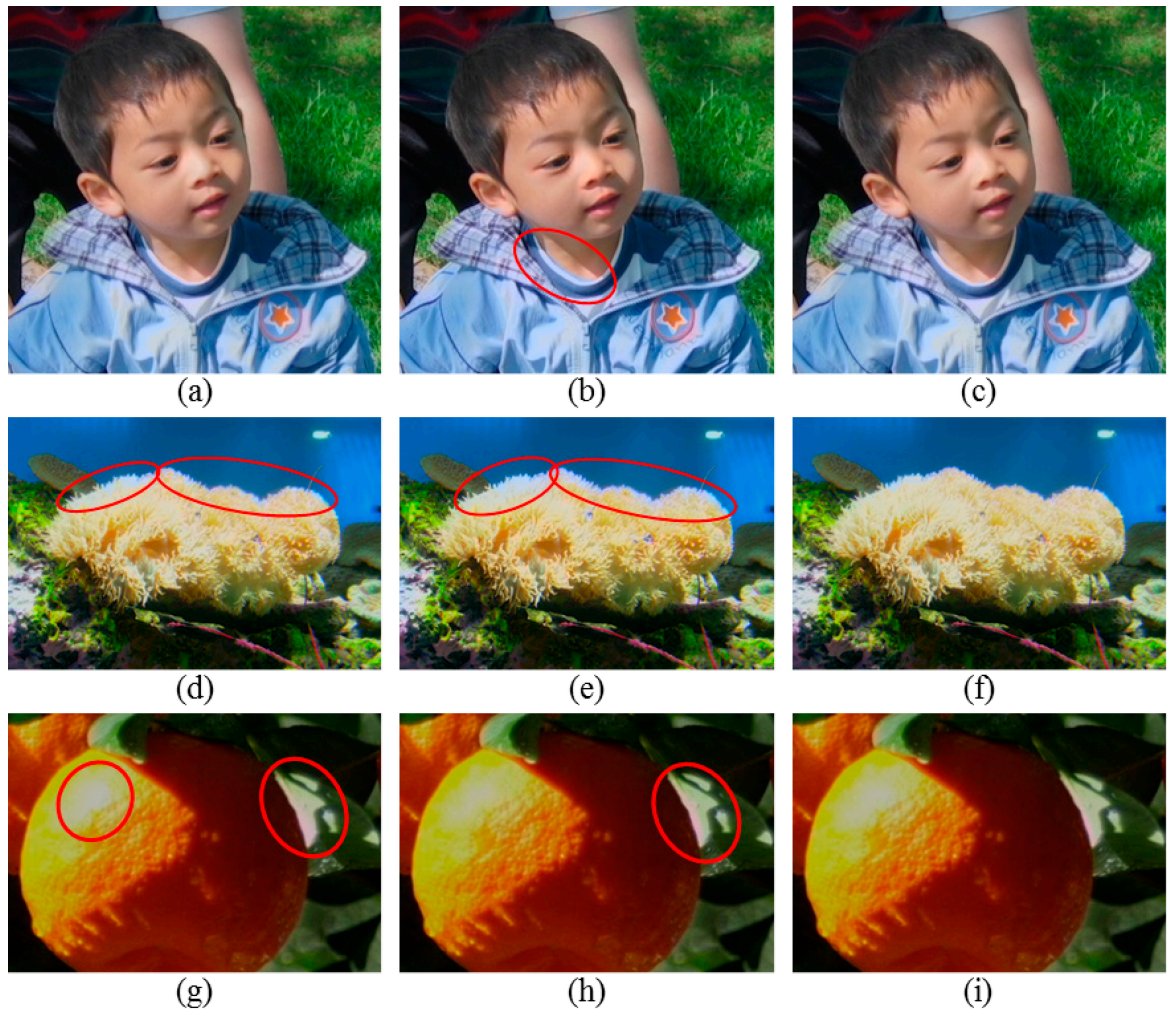
Comparison of correction results for an over-exposure. The OERs in (**a**), (**d**), and (**g**) were detected using the brightness thresholding method (Y ≥ 230) [9]. The OERs in (**b**), (**e**), and (**h**) were detected using the over-exposed map based thresholding method [10]. The OERs in (**c**), (**f**), and (**i**) were detected using the proposed method. All OERs were corrected using Guo's method [10]. The results are best viewed in the electronic version.

For the *Kid* image, since the over-exposed map based thresholding method over-detects the OER, as shown in Figure 5c, the color of the neck adjacent to the shirt is unnecessarily changed into a blue color after correction as shown in Figure 7b. However, when the brightness thresholding method or the proposed method is employed, the OERs are appropriately corrected, and the WERs are well preserved as shown in Figure 7a,c.

When the brightness thresholding method or the over-exposed map based thresholding method is utilized for the *Coral* image, the blue color of the water is incorrectly propagated into the top of the coral through the over-exposure correction, as shown in Figure 7d,e. In particular, although the right-top area of the coral in the original *Coral* image appears to be properly exposed, the WER is identified as the OER owing to the over-detection, as shown in Figure 5b,c, and is unnecessarily corrected. Thus, the quality of the corrected image is severely degraded as compared with the original image. On the other hand, the WER can be well preserved without improper correction when the correction is performed using the proposed detection method as shown in Figure 7f.

Since Guo's correction method decreases the brightness of both the WER and OER, the brightness of the perceived OERs in the *Oranges* image is decreased as shown in Figure 7g even though the OERs are not detected when the brightness thresholding method is utilized. However, its color correction result is almost same with the original image without notable improvement in quality. Because the over-exposed map based thresholding method detects the OER widely, the over-exposure generated on the orange is corrected more vividly compared with the other results, as shown in Figure 7h. However, in this result, the light orange color is severely propagated into the leaf region. In Figure 7i, the OER on the orange is corrected better than the result in Figure 7g and worse than the result in Figure 7h. Actually the difference of correction results among these three results is insignificant for the OER on the orange. However, the OER on the leaf is corrected most naturally when the OER is detected by the proposed method.

**Figure 8.**
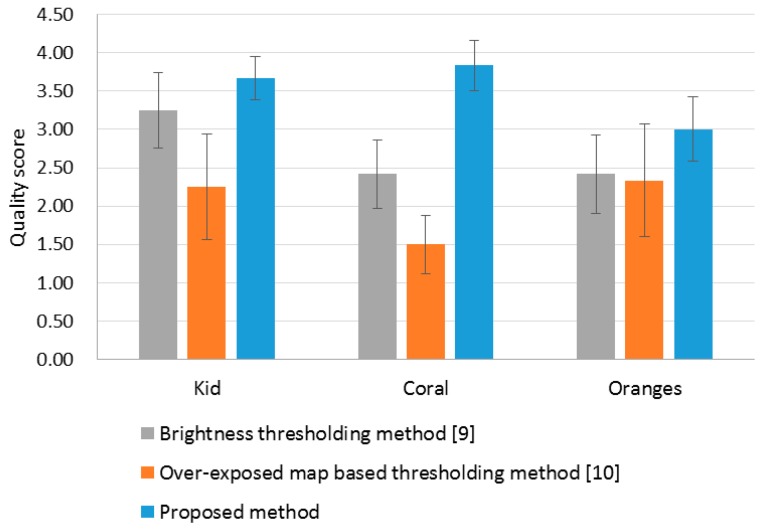
Subjective evaluation result for the over-exposure correction results.

Figure 8 shows the subjective quality evaluation result using the same experimental environment described in Section 4.1. When three corrected images were displayed simultaneously, the same subjects who participated in the experiment in Section 5.1 assessed the visual quality according to the grade scale in Table 1. The average subjective scores with 95% confidence interval, shown in Figure 8, demonstrate that the performance of the OER correction can be improved effectively by adopting the proposed OER detection method. It is clearly advantageous to use the proposed OER detection method for rendering visually pleasing images through the over-exposure correction.

## 6. Conclusions

In this paper, we observed that the results of conventional OER detection methods deviate considerably from the actual OER perceived by the human eye. To overcome this problem, we presented a new OER detection method for identifying the over-exposed pixels by thresholding the saturation value. Based on the characteristics of the saturation sensitivity of the HVS, as well as the results of our subjective experiment, we introduced a new function for adaptively determining the saturation threshold value depending on the color and brightness of each pixel. The experimental results show that the OER perceived by the human eye is successfully detected by the proposed method, which also influences the improvement of the OER correction performance.

## Figures and Tables

**Table 1.** Comparison scale for the subjective quality evaluation [24].

**Grade Scale**	**Quality**
5	Excellent
4	Good
3	Fair
2	Poor
1	Bad
